# Supplementary material for: Are carbon emissions trading and green financial instruments synergistic? -Comprehensive quantitative research based on content analysis
Source: PLoS One. 2024 Mar 7;19(3):e0298601. doi: 10.1371/journal.pone.0298601 (PMC10919601; doi:10.1371/journal.pone.0298601)
Supplement: S1 Data — (ZIP) [file pone.0298601.s001.zip › Data/地级市Z_F工作报告.zip!/╜¡╬≈/╨┬╙α╩╨/└┤╫╘╣1⁄2╓┌║┼í╛┬φ┐╦╩2╛▌═°í┐_2012.docx]

各位代表：

　　现在，我代表市人民政府向大会作政府工作报告，请予审议，并请市政协委员和列席会议的同志提出意见。

　　一、2011年工作回顾

　　过去的一年，在省委、省政府和市委的正确领导下，全市坚持以科学发展观为指导，积极应对宏观经济环境变化，特别是欧债危机的影响，各项工作迈上新台阶。我们迎来了新机遇，新余成为全国节能减排财政政策综合示范城市、国家“城市矿产”示范基地和资源枯竭转型试点城市，为加快发展注入了强劲动力；我们描绘了新蓝图，制定了国民经济和社会发展“十二五”规划纲要，凝聚了全市上下共识；我们实现了新跨越，全市人均生产总值突破1万美元，财政总收入突破100亿元，经济社会延续了健康发展的良好势头，多项主要经济指标增速继续保持全省前列，顺利实现了“十二五”良好开局。

　　经济实力进一步提升。全市生产总值779.2亿元，增长13.3%，人均生产总值10552美元。财政总收入111.3亿元，增长38％；其中，地方财政收入69.4亿元，增长38.9%。固定资产投资626.4亿元，增长28.2％。社会消费品零售总额132.8亿元，增长18.2%。出口总额20.7亿美元，增长3%。

　　发展后劲进一步增强。全年在建施工项目670个，其中新开工项目480个；新开工亿元以上项目100个，累计完成投资213.8亿元，其中工业项目68个，完成投资160.3亿元。100个重点项目97个在建，完成投资229.5亿元。新增16个省重点建设项目，争取省级用地指标7690亩。岳吉铁路途经新余、白梅水利枢纽工程立项取得实质性突破。

　　工业经济进一步壮大。规模以上工业增加值354.4亿元，增长19.2％；主营业务收入1580.3亿元，增长32.2％。一批重大工业项目快速推进，赛维1.5万吨硅料、江锂年产5000吨电解镍项目达产达标；新钢1550毫米冷轧项目竣工投产；江西华电3000台螺杆膨胀动力机生产线投产；力德风电500台兆瓦级永磁风力发电机、瑞晶四期20条电池片项目顺利推进。产业特色鲜明，三大产业主营业务收入1238亿元，占规模以上工业的78.4%，其中钢铁产业738亿元、新能源410亿元、新材料90亿元。集聚度不断提高，工业园区主营业务收入、工业增加值、利税分别为858亿元、212亿元、70.3亿元，增长27%、15%和25%，其中高新区主营业务收入583.6亿元，增长30.1%。龙头企业支撑作用强化，主营业务收入过亿元的企业150家，其中过5亿元企业42家；新钢、赛维进入中国企业500强。

　　现代服务业进一步繁荣。旅游业稳步增长。仙女湖佛教文化博览园、抱石文化创意园、分宜“中国洞村”、渝水区百丈峰景区、毓秀山国家森林公园景区、江西自然科学博物馆、袁河生态旅游等项目建设顺利推进，城市规划展示馆、工业展览馆相继建成。全市旅游总收入33.2亿元，增长31.3%。金融业加快发展。新组建小额贷款公司2家；新引进证券公司、保险公司各1家。社会融资总额突破1000亿元，其中，间接融资761.6亿元，增长22.7%；直接融资242.5亿元，增长38.7%。商贸流通业强力推进。大力实施商贸流通业“1020”工程，赣西万商红（国际）商贸物流中心快速推进；沃尔玛购物广场、赣西副食品批发市场、星辉鲜活农产品批发市场开工建设；腾达电器、步步高加快扩展，中国光伏大市场投入运营。

　　城乡统筹进一步推进。扎实开展统筹城乡经济社会发展一体化试点，编制完成经济发展战略规划，城市总体规划已经省政府批准。城镇化率达63.58%，提高1.99个百分点。城市建设力度加大。建成区面积67平方公里，扩大3平方公里。袁河生态新城核心区绿化景观工程基本竣工；孔目江新城核心区基本建成；毓秀山国际生态城一期工程全面开工；高铁新区各项规划和核心区城市设计已经完成，钟灵大道全面推进，站前广场开工建设；职教园区建设顺利启动，基础设施快速推进。启动了全国生态文明城市创建活动，城市绿化覆盖率提高到52.67%，人均公园绿地面积提高到17.39平方米，被评为全省生态园林城市。镇村建设成效明显。11个中心镇共安排重点项目119个，开工项目69个。小城镇建成区面积增加16平方公里，集镇人口增长8%，自来水普及率100%，绿化覆盖率34.6%。329个新农村建设点投入资金1.86亿元，重点打造了8个精品示范村，昌坊等6个村获全省“百家优美村庄”称号。城乡设施日趋完善。清宜公路货运通道竣工通车，仙女湖大道西延段、环城南路建设和樟排线水西至罗坊段改造加紧推进。洋坊编组站扩建工程建成投运，杭南长客专（新余段）征地拆迁完成。“农田水利基本建设年”活动扎实推进，完成农田基本建设工程10项，完成农村土地综合整治14.6万亩，31座小（二）型水库除险加固全面完成。农业基础夯实巩固。粮食生产面积153.9万亩，总产61.1万吨。新增高产油茶3.3万亩、花卉苗木2.6万亩，果业面积达22.8万亩、蔬菜播面22万亩。大力推进农业产业化“1010工程”，省级以上龙头企业达27家，全年完成销售收入59.7亿元，实现利润3.9亿元，带动农民增收4.4亿元。“恩达家纺”实现了我市中国驰名商标零的突破。

　　创新能力进一步提高。科技进步对经济增长贡献率达52%，被评为全国十大最具成长力创新型城市。大力推进科技创新。“543211”科技创新工程顺利推进。新余国家级高新技术产业开发区、新余国家现代农业科技园正式挂牌。先后与中国工程院、华南理工大学、天津大学等签订产学研战略合作协议。全市建有1个国家级高新区、2个国家级高新技术产业特色基地、3个国家级研发平台、5个省级研发平台、9个省重大高新技术产业化项目、7个省创新型试点企业和优势科技创新团队。新钢公司“高品质船板高效化制造关键技术研发与集成创新”课题获国家科技进步二等奖。大力抓好人才建设。选拔了首届10名市政府特殊津贴人员和第五批29名市学术技术带头人。组织开展了院士专家咨询服务活动，市政府院士顾问增至13名。新钢、赛维、力德三个中国工程院院士工作站建立。赛维博士后工作站正式授牌。8人被评为“赣鄱英才555工程”首批人选，入选人数列各设区市首位。人才公寓建设加紧推进。赛维成为全省首个国家引进国外智力示范单位。“人才引领产业、产业集聚人才”的做法被中组部向全国推广。大力发展教育事业。突出教育均衡发展，城乡义务教育各项免费、补助政策全面落实，“幼儿进园、小学进镇、中学进城”工程建设效果明显。全市职业院校在校生达9.47万人，万人以上职业教育院校4所。江西省职业教育园区已有6所院校进驻。现代学徒制试点得到教育部肯定。

　　改革开放进一步深化。各项改革不断深入。全省七个系统国有企业改革工作现场推进会在我市召开。新余日报社、市电台、市电视台、市歌舞剧院改革全面完成。医药卫生体制改革稳妥推进，积极探索公立医院改革试点，基本药物制度全面覆盖政府举办的基层医疗卫生机构。城乡一体化户籍管理制度改革扎实推进。招商引资力度加大。全年实际利用外资5.94亿美元，增长11.8%；实际引进内资325.9亿元，增长35%；利用省外5000万元以上项目实际进资188.9亿元，增长34.4%。央企对接进一步深化，与北京石景山区签订了战略框架协议，与中国保利集团的合作迈出实质性步伐，中国建材集团300万吨矿渣微粉项目等央企投资项目落户我市。积极推动企业增资扩股，283户企业增资扩股额达52.9亿元。外贸出口逆势而上。出口总额居全省第五位。共组织24家外贸企业参加国内展9次，27家企业参加境外展21次。推动24家外贸企业加入电子商务平台。新增外贸出口备案登记企业76家，其中新增出口实绩企业34家。非公经济贡献凸显。全年新增个体工商户6711户、私营企业1374户，非公经济增加值占生产总值的比重提高到64.3%，上缴税收占财政总收入的63%，荣获全省“发展非公有制经济先进设区市”称号。

　　人民生活进一步改善。城镇居民人均可支配收入19719元，增长13.6％；农民人均纯收入8813元，增长17.9％。各级财政民生事业支出38亿元，全面或超额完成省政府下达的93项民生指标、市政府确定的100项民生工程和10件实事。全年新增城镇就业3.08万人，新增转移农村劳动力2.12万人；“招工、就业、培训”三位一体工作模式在全省推广。在全省率先实现养老保险、城乡基本医疗保险和居民最低生活保障全覆盖。城乡低保标准每人每月分别提高到320元和130元，城镇职工月人均基本养老金标准高出全省平均水平80元。在全省率先建立社会救助和保障标准与物价上涨挂钩联动机制，率先为低收入群体发放临时物价补贴。基本完成2020套廉租住房建设，发放廉租住房租赁补贴4803户；开工建设经济适用住房265套。7个棚户区改造项目全面开工，完成改造面积35.49万平方米。按照每个社区不少于320平方米建设标准，全面落实社区办公活动用房。严格落实大中型水库移民后期扶持政策，发放移民直补资金1959万元。

　　社会事业进一步发展。文化事业不断繁荣，喜获“全国文化体制改革工作先进地区”称号，成功举办庆祝建党90周年大型纪念活动等多项文化活动，广播剧《老镜子》获全国广播剧专家奖金奖和中国广播影视大奖提名奖，少儿舞蹈《荷塘乐》首次走出国门，应邀赴维也纳演出。罗坊镇和昌坊村荣获全国文明村镇，市地税局、市国土资源局、市中行、市房管局、新余海关、市人行荣获全国文明单位。卫生服务能力全省先进，全市新农合参保人数72.35万人，参保率达98.05%；完成免费白内障复明手术437例、唇腭裂手术7例，完成儿童白血病免费救治13例、先天性心脏病免费救治181例。全面完成节能减排任务，主要污染物减排工作受到省政府表彰。省市共建全国食品药品安全示范城启动，主要食品品种检测指标合格率97.56%。计生工作被列为“全国流动人口计划生育基本公共服务均等化”和“创建幸福家庭”两个国家级试点。承办“七城会”相关赛事受到省里表彰。全国双拥模范城创建扎实推进。档案工作获全国先进。安全生产形势总体稳定。加大矛盾纠纷排查调处力度，调处率达92%；推进以群众工作统揽信访工作试点；加强社会治安综合治理，影响公众安全感的八类重点案件发案率下降32.5%，公众安全感测评满意率连续四年上升，切实维护了国家安全和社会稳定。统计、物价、人事编制、审计、工商、海关、检验检疫、质监、新闻出版、民族宗教、外事侨务、涉台事务、驻外机构、机关事务、防震减灾、妇女儿童、方志、科普、气象、人防、证券保险、邮政通信、烟草、海事、拥军优属、国防动员、民兵预备役、社科研究、红十字会人道援助和社会慈善、残疾人及老龄等工作取得新成绩。

　　政府建设进一步加强。全面推进依法行政，法治政府建设得到加强。扎实开展“发展提升年”活动，不断提高行政效能，切实转变工作作风，高度关注事关群众切身利益的热点难点问题。推进行政审批制度“两集中两到位”改革工作，开展行政审批大提速活动，行政审批效率进一步提高。加强网上审批和电子监察系统建设，所有行政审批事项纳入网上监察，进一步规范了审批程序。推进政务公开，积极完善各类公开办事制度，不断提高政府工作透明度。进一步精简会议和文件。

　　各位代表，过去的一年，我们之所以能够战胜各种困难，取得这些成绩，是省委、省政府和市委正确领导的结果，是全市人民共同奋斗的结果。在此，我代表市人民政府，向全市广大工人、农民、知识分子、干部和历任老领导、老同志，向各民主党派、工商联、无党派和社会各界人士，向驻市部队、武警官兵和公安干警，向所有关心、支持新余发展的国内外朋友表示衷心的感谢并致以崇高的敬意！

　　在肯定成绩的同时，我们也清醒地认识到，经济社会发展还面临不少困难和问题。全市经济总量偏小，城乡居民收入偏低，发展不足仍然是我们面临的主要矛盾。经济发展与环境承载能力的矛盾、工业经济快速发展与第三产业滞后的矛盾、财政收入增长与民生事业支出增加的矛盾、改革深入推进与群众利益诉求增多的矛盾、加快发展与一些行政机关工作人员能力素质不相适应的矛盾更加凸显。对于这些问题，我们一定高度重视，下决心认真加以解决。

　　二、2012年主要工作

　　今年是实施“十二五”规划承上启下的重要一年，是贯彻落实省市党代会精神的关键一年，做好今年的各项工作至关重要。今年政府工作的总体要求是：坚持以科学发展观为指导，深入贯彻落实党的十七届六中全会、中央经济工作会议和省第十三次党代会、市第七次党代会精神，牢牢把握经济发展“稳中求进、稳中求快”的工作基调，紧紧围绕建设和谐富裕文明新余的奋斗目标，积极融入鄱阳湖生态经济区、加快建设国家新能源科技城，切实转变经济发展方式，加快经济结构调整，大力实施工业立市、科教兴市、人才强市战略，深化改革开放，优化生态环境，保障改善民生，提升经济竞争力、科技创新力、区域辐射力、文化影响力、环境吸引力、社会保障力，促进经济平稳较快发展和社会和谐稳定。按照上述要求，全市经济社会发展的主要预期目标是：生产总值920亿元，增长13.2%；财政总收入133.5亿元，增长20%，地方财政收入80.6亿元，增长16%；规模以上工业增加值425亿元，增长20%，主营业务收入2000亿元，增长26.6%；固定资产投资783亿元，增长25%；外贸出口总额22亿美元，增长6%；社会消费品零售总额155.4亿元，增长17%；城镇居民人均可支配收入22480元，增长14%，农民人均纯收入10047元，增长14%；社会融资总额1160亿元，增长15.5%。居民消费价格指数控制在4%左右。

　　为实现上述目标，重点抓好以下几方面工作：

　　（一）以积极融入鄱阳湖生态经济区、加快建设国家新能源科技城为主线，全力推动经济平稳较快发展

　　加快推进工业经济发展。坚持工业立市不动摇，全力推进“十百千亿”工程，大力实施设备更新、技术革新、品牌创新“三新工程”，推进工业化和信息化“两化融合”，做大做强战略性新兴产业。抓好支柱产业发展。新能源产业要积极推进光伏产业向下游及应用产品延伸，推动锂电产业跨越发展，加快形成产业集群。重点抓好赛维2000兆瓦硅片和1000兆瓦电池片、瑞晶800兆瓦电池片、升阳540兆瓦电池片、吉阳400兆瓦电池片、海宸光电200吨高纯砷和200万片砷化镓、赣锋锂业1万吨锂盐、环明新能源1万吨磷酸铁锂等项目建设。新材料产业要大力发展金属新材料、无机非金属新材料和高分子复合新材料等产品，提高产业规模和经营水平。重点抓好江锂2万吨电解镍和12万吨氧化镁及28万吨白炭黑、江镍1万吨镍丝镍带、沃格光电240万片玻璃薄化、东鹏新材料1000吨氟化锂和500吨碳酸铯、旭扬新能源144万毫米蓝宝石晶棒和120万片蓝宝石衬底、铂砾耐200万平方米建筑模板和15万套沼气池等项目建设。钢铁产业要加快转型升级，增强新产品开发和品牌创建能力，促进产业向下游延伸，大力发展机械加工业，提高钢铁产品附加值。重点抓好新钢恒基25万吨彩钢板和72万吨镀锌板、新钢良矿扩界工程及太平山矿区深部开采、贝卡尔特（新余）2.3万吨镀锌钢丝、永盛矿冶1.3万吨钒铁、新特20万吨汽车板簧、运良锻压6万吨精密锻件、嘉锐工贸40万吨废钢加工等项目建设。新能源、新材料、钢铁三大产业分别完成主营业务收入480亿元、100亿元和900亿元。抓好工业园区建设。高新区重点对接新能源产业，经济开发区对接钢铁和机械加工产业，分宜工业园对接新材料和动力与储能电池产业，引导新上工业项目按园区产业功能定位落户。推进园区标准厂房建设，提高集约用地水平。高新区要加快推进三个“区中园”建设，抓好园区管理体制创新，试行企业化管理，提高管理水平，加快进入中部地区园区前列。经济开发区要整合资源、合理规划，力争升格为省级工业园区。分宜工业园要抓好新材料、动力与储能电池、城市矿产和机械加工制造四大产业发展。高新区、分宜工业园、经济开发区规模以上工业主营业务收入分别达800亿元、200亿元、160亿元以上。抓好优强企业培育。落实扶持政策，引导各类要素向优势企业和特色产业集中，优先解决优势企业在发展过程中遇到的困难和问题，推动企业裂变扩张。新增主营业务收入过5亿元企业5户，培育过500亿元、400亿元和100亿元企业各1户。

　　加快推进项目建设。抓紧重大项目争取。围绕国家产业政策，做好项目的筛选、编制、申报争取工作。利用我市被列为全国节能减排财政政策综合示范城市、国家“城市矿产”示范基地、资源枯竭转型试点城市的有利条件，做好规划编制和项目申报工作。积极跟进对接白梅水利枢纽工程、大唐国际新余发电公司2台66万千瓦机组、仙女湖湖泊生态保护国家试点等事关新余长远发展的重大项目。抓实重点项目建设。计划安排投资2000万元以上大中型项目221个，总投资1660亿元，年度计划投资436亿元，建成投产项目79个。坚持每季度开工亿元以上项目25个，集中力量竣工一批重大项目。全力配合推进岳吉铁路（新余段）建设。抓好推进机制落实。强化和完善“六个一”推进机制，严格实行重点项目建设目标管理考核办法，定目标、定时间、定措施、定责任，确保项目按进度推进。实行统一调配制，由责任单位牵头定期组织指导协调。实行督办考核制，将指导帮扶工作纳入督查范围，列入全年综合目标考核。

　　加快推进现代服务业发展。切实把加快现代服务业发展作为调整优化全市经济结构的突破口，推动商贸流通业“1020工程”项目早见成效。大力发展商贸业。完善提高中国光伏大市场，加快腾达电器、步步高商业网点建设，力争赣西万商红（国际）商贸物流中心、星辉鲜活农产品批发市场、赣西副食品批发市场实现营运，开工建设新余国际商城、大欢乐购物中心，争取新汽车大市场（九鼎）年内开工。大力发展物流业。开工建设中国江西海峡现代物流园、赣铁物流园，加快推进穗东物流中心建设，确保正大钢材交易市场、林安物流中心竣工开业。加快推进物流公共信息平台建设。抓好汽车货运业发展，力争道路货物运力增长10%。大力发展金融业。新组建1家村镇银行，力争新设立1家股权投资基金公司、2家小额贷款公司、1家担保公司。力争赛维硅料、瑞晶太阳能年内上市，做好永盛矿冶上市申报工作。支持上市公司通过增发、配股等方式实现再融资，支持企业利用资本市场和银行债券市场实行发债融资。争取年末贷款余额突破730亿元、企业上市等直接融资余额突破280亿元。大力发展旅游业。启动仙女湖5A级景区创建工作，重点抓好佛教博览园、体育公园二期、抱石文化创意园等项目续建，抓好商务、休闲配套设施建设；加快毓秀山国家森林公园建设，推进“中国洞村”、袁河观光游、孔目江湿地公园三期、百丈峰景区等旅游项目建设。大力发展红色旅游、工业旅游、休闲农业和乡村旅游。实现国内旅游收入增长30%以上。

　　加快推进开放型经济发展。深化重点领域改革。完善市县（区、管委会）财政管理体制，支持财税部门依法加强征管；继续深化医药卫生体制改革，积极稳妥推进公立医院改革，在分宜县人民医院开展取消药品加成试点工作；深化行政管理体制改革，改进政府调控经济方式，理顺部门管理职责；分类推进事业单位改革，全面落实机构编制实名制管理；做好中小学教师职称制度改革试点工作；健全国有资产监管体制，实施国有资本经营预算；深化农村经济体制改革，加快农村集体土地确权登记发证工作，建立健全农村土地流转有形市场体系；积极推进户籍管理制度改革；抓好国有林场改革，持续深化集体林权制度改革；扎实做好企业一套表统计改革实施工作。加大招商引资力度。坚持算大账不算小账，算长远账不算眼前账，对投资额度大、产业带动力强的大项目、好项目敢于让利，采取一事一议、一企一策的办法，尽最大可能、以最优惠政策吸引项目落户。创新招商方式，完善政府搭台、县区唱戏的招商模式；坚持领导招商，推动重大项目引进落户；完善专业招商，强化专业招商小分队作用；实行委托招商，注重发挥商会、行业协会等中介组织作用；突出重点招商，围绕台湾、长三角、珠三角、闽东南、长株潭等重点地区，新能源、新材料、钢铁、机械制造等重点产业，开展招商推介活动；对接央企招商，力争全年引进央企项目6个以上。坚持把企业增资扩股作为招商引资的重要方式来推动，一手抓招商引资，一手抓增资扩股，实行双轮驱动。全年实际利用外资6.83亿美元，增长15%；实际引进内资423亿元，增长30%，其中引进省外5000万元以上项目资金226.7亿元，增长20%。积极扩大外贸总量。扶持外贸企业开拓市场、降低贸易风险，优先安排重点出口企业申报项目。推动各县区建立健全外贸服务机构。进一步发展铁海联运，完善铁路口岸作业区，力争开工建设公路口岸作业区，推动电子口岸建设与应用。积极申报和建设国家出口加工区。

　　加快推进农业产业化。抓好粮食生产，稳定粮食种植面积150万亩、总产量60万吨，保障大宗农产品有效供给。大力推进 “1010工程”，重点扶持以珊娜果业为龙头的15万亩新余蜜桔基地、以恩达家纺为龙头的10万亩优质高效苎麻基地、以天欣源为龙头的10万亩高产油茶基地、以阳光集团为龙头的20万亩花卉苗木基地、以艾格菲为龙头的30万头生态养猪产业基地建设，大力推进光伏大棚有机蔬菜基地建设，新增新余蜜桔1万亩、高产油茶2万亩，确保花木面积达到5万亩以上。

　　加快推进实体经济发展。加强政策引导和扶持，重点支持符合产业政策的实体经济发展。大力发展非公有制实体经济，以“总量倍增、扶优培强、产业集聚、政策创优、服务提升”五大工程为重点，推动全民创业，促进各种所有制经济公平竞争、共同发展。力争非公有制经济增加值占生产总值的65%以上，上缴税收占财政总收入的68%以上。

　　（二）以建设全国节能减排财政政策综合示范城市为主线，全力推进生态文明建设

　　推进城乡一体化。扎实开展生态文明城市建设，着力打造全国节能减排财政政策综合示范样板城市，启动中国人居环境奖、全国节水型城市创建活动。全面推进新型城镇化和统筹城乡一体化建设，城镇化率提高2个百分点。完善城乡规划。以城市总体规划为指导，全面完成主城区控制性详细规划和各专项规划编制，做到城市规划区内用地控规全覆盖。完成乡级土地利用规划编制。做好重点区域、重点地段、重要节点城市规划和风貌设计。提高乡村规划编制水平，修编完善11个中心镇总体规划和示范镇控制性详规。做精做美主城区。按照“改造老城区、建设生态新区、提升高新区”思路，将城市建设重心由外延扩张向内涵提升转变。加快“三城二区”建设，推进袁河生态新城国家低碳试点，确保抱石大道延伸段、景观绿化工程等全面完工，加快安置小区建设；完善孔目江新城建设；加快推进毓秀山国际生态城低碳节能小区建设；全面完成高铁新区钟灵大道、站前广场、车站站房建设，加快圣地花园拆迁安置小区建设；职教园区交通网基本成型，基础设施逐步完善。加快市民服务中心建设。抓好以四眼井、观下等为重点的棚户区改造。加强城投、交投、公投等融资平台建设；搞好城市土地经营，实现年内收储土地2000亩，规划储备1.2万亩。抓好新增建设用地报批，力争报批1万亩。创新城市管理。完成“数字新余”地理空间框架建设，实行数字化城市管理，推进“城市管理社区化、社区管理物业化、物业管理市场化”，落实城管分片包干责任，完善城乡道路安全设施，强化城管执法监督，全面提升城市管理水平。加快城乡公路建设。推进城乡公路路网改造，做好石镇线（上高至新余）一级公路改建工程前期准备工作并力争年内开工，加快推进樟排线新余至罗坊段、余新公路、经开大道建设，全面完成仙女湖大道东延段、五一南路南延改造工程和滨江路、环城南路、仙女湖大道西延段建设，规划重载车专用通道，着力构建“一环二环三环”为主干道的城市交通网，加快主城区至周边县一级公路和乡镇之间二级公路建设。狠抓中心镇建设。完善基础设施，重点抓好总投资27亿元的54个项目建设和集镇农民社区建设。培育特色产业，加快形成 “一镇一产业一特色”格局。探索启动市直管重点镇试点，按照管理城市的要求管理乡镇。创新投融资体制机制，将财政扶持资金改为注入担保机构，通过市场办法，拓展资金来源。推进城乡公共服务均等化，完善医疗、教育、敬老院等公共服务设施，健全社会保障体系，实现医保、低保、农村养老保险等社会保障体系全覆盖。抓好新农村建设。重点打造清宜公路新农村建设精品示范线，建设280-300个新农村点，新建8个精品村。推进百万树木进村入户，完成新农村建设点植树20万株。推行垃圾处理新模式，完成85%以上自然村的整治任务，基本实现农村环卫保洁达标和垃圾集中处理。加强农田水利基本建设，抓好袁河（孔目江）生态水利水电工程完善和孔目江西堤茶山段防洪堤建设，完成分宜城西防洪工程建设。完成45座小（2）型病险水库除险加固，启动刘家水闸和袁惠渠灌区6座大中型病险水闸除险加固工作。抓好分宜小农水重点县建设。整合资金建设高标准农田3万亩以上。

　　发展低碳经济。推进经济文明与生态文明融合发展，加快形成低能耗、低污染、低排放的低碳产业体系。抓好国家“城市矿产”示范基地建设，积极引进企业入驻，力争基地销售量300万吨以上，销售额120亿元。加快建设太阳能光伏电站、光电一体化标志性建筑及新能源综合利用示范小区，实施光伏发电应用30兆瓦、光热建筑应用465万平方米、公共建筑节能改造100万平方米、10个新能源综合利用示范小区建设，培育3至5个绿色建筑示范小区。发展低碳交通，打造以公共交通为导向的低碳交通体系。倡导低碳生活，开展绿色节能低碳机关、学校、家庭创建活动。

　　深化节能减排。严格执行节能目标责任制，督导重点用能企业落实节能目标。制订节能监测管理体系，推行项目建设节能评估和审查制度。加大落后产能淘汰力度，确保按时间节点关停减排计划企业，综合运用结构减排、工程减排、管理减排三大措施，全面完成省政府下达的主要污染物减排任务。

　　加强生态保护。扎实开展环境污染治理活动，截断新污染源，提高审批企业环境准入门槛，严禁污染企业进入；治理污染源头，对江河流域，按照省政府规划要求治理，对企业污染，由企业自行负责，做出计划，分年度治理。积极支持新钢建设花园式工厂。推进森林城市建设，继续抓好造林绿化“一大四小”工程，确保完成8万亩建设任务；积极开展“身边增绿”、“拆墙透绿”行动，加强生态园林、景观带、公共绿地建设；坚持“在保护中利用、在利用中保护”，将毓秀山建成城中森林公园，巩固国家森林城市创建成果，主城区绿地率、绿化覆盖率、人均公园绿地面积分别达到51.96%、53.37%、18.45平方米。加强生态公益林管理，启动市级生态公益林。创建生态县区、生态乡镇、生态村，加快孔目江流域生态治理和产业结构调整，率先实施生态补偿机制。

　　（三）以实现人均期望寿命80岁为主线，全力促进社会和谐

　　推进科教兴市。发挥科技示范作用。全面实现“543211”科技创新工程各项目标。做好新能源科技城规划编制并组织实施。提高企业自主创新能力，加快建立企业主导产业技术研发创新的体制机制，加强重点产业技术攻关，支持企业承担国家和地区重大科技项目，鼓励规模以上工业企业普遍建立研发机构。加强科技创新平台建设，抓好现有国家和省级创新平台建设，大力提升国家光伏工程技术研究中心、国家稀土永磁电机技术研究中心等国家、省级研发平台科技创新能力；鼓励和支持企业积极申报建设国家及省级科研中心，推动国家动力与储能电池高新技术产业化基地、国家新能源检测实验室获批。推进科技与产业结合，加快传统产业改造升级，实现战略性新兴产业突破，增强产业技术优势，力争三大产业最新科研成果尽早在我市孵化。推进产学研一体化，以技术对接为重点，以共同设立技术研发中心为载体，建立产学研对接长效机制，加强与国内外著名高校、科研机构的科技合作，大力引进科研机构，争取华南理工大学新余工业技术研究院挂牌。加大科技投入，形成多元化科技投入机制。做好创建国家知识产权示范城市工作，确保专利申请量和授权量两项指标均增长20%以上。重视人才队伍建设。坚持以人才引领产业、以产业聚集人才，大力实施“十百千万”工程，着力引进科技创新人才、企业管理人才和海外高层次人才。加大人才政策和机制创新力度，努力在职称评聘和高层次人才评选表彰等方面有重大创新，加快推进人才管理改革试验区建设，完成高层次人才公寓建设并投入使用。加快教育事业发展。全力推进教育体制改革试点、教育投入和管理、学前教育发展三项重点工作，确保财政教育支出占公共财政支出比例达11%。大力推进中小学“班班通”工程，全面推进幼儿园规范化建设。推进职教园区建设，抓好现代学徒制试点，争取新入驻2所学校。支持新余学院加快建立现代大学体制机制，为建成江西工学院奠定坚实基础。

　　办好十件实事。⑴推进廖家江、贯早江环境治理。⑵打通公园路、长青南路等城区“断头路”，提升公共交通服务水平。⑶建成渝水六小，加快规划并开工建设渝水五小、江西二化安置小区学校，加快北京师范大学新余附属学校建设。⑷开工建设保障性住房、棚户区改造住房共6225套（户），竣工5901套（户），其中，新建廉租住房1230套、公共租赁住房1620套。⑸新增5000平方米以上专业市场或超市2-3家，升级改造农贸市场3个。⑹推进“农村户户通自来水工程”，新增6万农民用上自来水。⑺抓好菜篮子工程，新增商品蔬菜基地8000亩。⑻完成农村公路建设137公里。⑼实施农村义务教育阶段学生营养餐试点。⑽全面完成主城区民用天然气置换。

　　保障改善民生。努力扩大就业规模，新增城镇就业人员2.16万人、转移农村劳动力1.35万人。不断扩大社会保险覆盖面，重点推进工业园区、非公有制企业职工、农民工、灵活就业人员等群体参加社会保险，城镇基本养老保险参保缴费人数17.21万人，征缴总额8.5亿元。完善临时救助、医疗救助和医疗保险同步结算制度，进一步提高城乡低保标准。抓好扶贫开发工作，坚持新增财力和公共资源重点向贫困地区倾斜。推进基本养老服务体系建设。启动新一轮敬老院改造提升工程。强化市场监管和价格检查，努力保持物价基本稳定。巩固和完善新农合制度，确保新农合参合率稳定在98%以上；健全国家基本药物制度；加快提升卫生服务能力水平，优先建设发展县级医院，巩固提升已达标的乡镇卫生院和村卫生室，抓好乡镇卫生院债务化解，推进社区卫生服务机构规范化、标准化建设。组织实施好“光明·微笑”工程、困难尿毒症患者免费血透等重大公共卫生服务项目。继续抓好水环境综合治理，新建雨污分流管网5公里、污水收集管网10公里，确保仙女湖、孔目江主要饮用水源地保护区水质稳定在国家地表水Ⅱ类标准以上，袁惠渠、袁河水质稳定在Ⅲ类标准以上，主要河流断面优质水率达100%。加强空气污染防治，确保城区空气质量稳定达到国家二级标准。抓好全国食品药品安全示范城创建工作，进一步提升食品药品安全水平。为实现人均期望寿命达80岁打下坚实基础。

　　创新社会管理。完善党委领导、政府负责、社会协同、公众参与的社会管理格局，建立政府调控机制与社会协同机制互联、政府行政功能与社会自治功能互补、政府管理力量与社会调解力量互动，公众依法理性有序参与的社会管理网络。完善矛盾排查调处机制，实现矛盾纠纷化解常态化、制度化。用群众工作统揽信访工作，落实信访工作责任制，完善领导干部定期接访、下访制度，教育和引导诉求方依法有序理性表达诉求。加强基层平安创建，建设和谐社区，科学合理调整社区规模，确保每个社区有较好的办公用房和工作条件。建立重大工程项目建设和重大政策制定社会稳定风险评估机制。加强流动人口、特殊人员管理服务。

　　发展社会事业。积极创建省级文明城市，为创建全国文明城市奠定坚实基础。加快文化基础设施建设，建设市文化中心，率先建好村农家书屋、乡镇文化站；争创全国公共文化服务体系示范区；深化文化体制改革，出台文化产业发展规划，制定有利于文化产业发展的优惠政策，推动文化产业加快发展。做好参加省第四届全民健身运动会组织工作，举办好市第七届运动会。抓好“全国人口和计划生育综合改革示范市”建设，确保“全国流动人口计划生育基本公共服务均等化试点”顺利完成任务，力争成为全国创建幸福家庭示范市。全面实施“六五”普法规划。促进哲学社会科学、广播影视、新闻出版、档案方志事业发展。加强国防教育、国防动员、国防后备力量和人民防空建设，增强军政军民团结。贯彻落实党的民族政策和宗教工作基本方针，依法管理宗教事务。保护妇女、未成年人合法权益，大力发展慈善、老龄、红十字会和残疾人事业。认真做好统计、外事侨务、防震减灾、海关、检验检疫等工作。

　　维护社会稳定。加强社会治安综合治理，推进平安新余建设，加强隐蔽战线斗争，严密防范、依法打击敌对势力的渗透破坏活动和各种违法犯罪，提高公众安全感。加大公共安全投入，建立安全生产长效机制，完善应急管理机制，健全事故灾难尤其是食品安全、消防安全、道路交通安全事故预警和应急处置体系，有效遏制重特大事故发生。

　　（四）以提高政府建设科学化水平为主线，全力打造人民群众满意政府

　　提高行政效能。深入开展“服务发展、创先争优”活动，全面落实岗位责任制、服务承诺制、责任追究制等一系列效能制度，推行“一线工作法”，形成领导在一线服务、干部在一线创业、问题在一线解决、成效在一线体现、形象在一线树立的工作格局。集中整治影响发展环境的突出问题，着力解决行政机关一些工作人员作风上的“庸、懒、散”和“假、浮、蛮”，为政不廉的“私、奢、贪”，以及行政审批中的低效率等问题，最大限度简化办事程序，缩短办事时限，打造一流的发展环境。

　　推进依法行政。健全行政管理体制，不断提高依法行政水平。完善决策程序，坚持公众参与、专家论证和政府决策相结合。规范行政执法行为，严格行政执法程序，做到公开、公平、公正、有序。自觉接受市人大及其常委会的法律监督、市政协的民主监督，认真听取各民主党派、工商联、各人民团体和各界人士的意见，加强监察、审计专门监督，高度重视新闻舆论监督、人民群众监督等社会监督。

　　加强廉政建设。健全拒腐防变教育机制、行政权力运行监控机制和反腐倡廉制度体系，加强建设工程招投标、财政转移支付、土地和矿产资源使用、政府采购、国有资产转让等公共资源管理权力的监管，强化工程建设领域项目信息公开和诚信体系建设，防止国家利益部门化、部门利益个人化。严格控制各项支出尤其是“三公”经费支出，确保每一项重大投资都符合国家政策，每一笔资金都用在刀刃上。

　　各位代表，做好今年的工作，责任重大、任务艰巨。让我们更加紧密地团结在以胡锦涛同志为总书记的党中央周围，在市委的正确领导下，深入贯彻落实科学发展观，大力弘扬“开放、创新、争优”的新余精神，进一步解放思想，奋发图强，扎实工作，奋力建设和谐富裕文明新余，以优异成绩迎接党的十八大胜利召开。
